# A randomized phase II trial comparing chemoimmunotherapy with or without bevacizumab in previously untreated patients with chronic lymphocytic leukemia

**DOI:** 10.18632/oncotarget.13412

**Published:** 2016-11-16

**Authors:** Neil E. Kay, Paolo Strati, Betsy R. LaPlant, Jose F. Leis, Daniel Nikcevich, Timothy G. Call, Adam M. Pettinger, Connie E. Lesnick, Curtis A. Hanson, Tait D. Shanafelt

**Affiliations:** ^1^ Mayo Clinic College of Medicine, Rochester, MN, USA; ^2^ Mayo Clinic College of Medicine, Scottsdale, AZ, USA; ^3^ Essentia Health's East Region, Duluth, MN, USA

**Keywords:** CLL, chemoimmunotherapy, bevacizumab

## Abstract

Bevacizumab is a monoclonal antibody targeting vascular endothelial growth factor (VEGF) with *in vitro* pro-apoptotic and antiangiogenic effects on chronic lymphocytic leukemia (CLL) cells. As monotherapy in patients with CLL, it has no clinical activity. Here we report the results of an open-label, randomized phase II trial comparing the combination of pentostatin, cyclophosphamide and rituximab (PCR) either without or with bevacizumab (PCR-B) in previously untreated CLL patients. A total of 65 evaluable patients were enrolled, 32 receiving PCR and 33 PCR-B. A higher rate of grade 3-4 cardiovascular toxicity was observed with PCR-B (33% *vs*. 3%, *p* < 0.003). Patients treated with PCR-B had a trend for a higher complete remission (CR) rate (54.5% *vs* 31.3%; *p* = 0.08), longer progression-free survival (PFS)(*p* = 0.06) and treatment-free survival (TFS)(*p* = 0.09). No differences in PFS and TFS by *IGHV* mutational status were observed with the addition of bevacizumab. A significant post-treatment increase in VEGF levels was observed in the PCR-B arm (29.77 to 57.05 pg/mL); in the PCR-B arm, lower baseline CCL-3 levels were significantly associated with achievement of CR (*p* = 0.01). In conclusion, the addition of bevacizumab to chemoimmunotherapy in CLL is generally well-tolerated and appears to prolong PFS and TFS.

## INTRODUCTION

Although chemoimmunotherapy (CIT) has substantially improved response rates, treatment free survival, and overall survival in patients with chronic lymphocytic leukemia (CLL)[1, 2], only 40-50% of patients achieve a complete remission (CR) and the majority have residual disease when evaluated using sensitive assays.[3] Approaches to both improve CR rates and reduce residual disease post CIT may reside on treatment strategies that modify the well-known positive influence on CLL B cell survival exerted by the microenvironment.

Interactions with various nurturing environments can enhance CLL B-cell resistance to apoptosis.[4] These interactions include cytokine mediated pro-survival signals by angiogenic molecules, such as vascular endothelial growth factor (VEGF) and basic fibroblast growth factor (b-FGF), which nurture CLL B-cells and promote the survival of CLL B-cells in part through up regulation of anti-apoptotic proteins.[5–7] In fact, both CLL B-cells and marrow stroma produce pro-angiogenic molecules, including VEGF and b-FGF, which can act in an autocrine or paracrine fashion to enhance leukemic B-cell resistance to apoptotic cell death.[8–11]

Bevacizumab is a monoclonal antibody targeting VEGF and has been shown to play a pro-apoptotic and anti-angiogenic effect on CLL cells *in vitro*.[12, 13] As a monotherapy, no significant clinical activity was observed with bevacizumab in patients with relapsed refractory CLL.[14] Multiple studies in solid tumors, however, suggest that bevacizumab has its greatest effect in combination with chemotherapy. [15–17] Higher VEGF levels have also been shown to predict less favorable outcomes among CLL patients receiving CIT, providing further rationale for testing the effect of anti-VEGF therapy in combination with CIT for patients with CLL.[18] Here we conducted a randomized phase 2 CIT trial where we used pentostatin, cyclophosphamide, and rituximab with (PCR) with an anti-VEGF agent, bevacizumab (PCR-B), or without bevacizumab (PCR) for patients with progressive but previously untreated CLL.

## RESULTS

### Baseline characteristics

The study opened in January 2009 and completed accrual of 68 patients in January 2013. Thirty-five patients were treated with PCR-B and 33 patients were treated with PCR. Of the 68 patients accrued, 3 were not evaluable (PCR-B: 2, PCR: 1) due to incorrect diagnosis, treatment on the wrong study arm, and withdrawal prior to receiving treatment, and were excluded from all analyses. Baseline characteristics of the 65 patients evaluable for the primary endpoint (PCR-B: 33, PCR: 32) did not differ significantly between the 2 arms (Table 1).

**Table 1:** Baseline characteristics

		PCR-B (Arm A) (N=33)	PCR (Arm B) (N=32)	*p*-value
Age [median (range); years]	65 (43 −81)	62.5 (50.0-78.0)	0.30
Male	20 (60.6%)	23 (71.9%)	0.43
ALC [median (range); x10^9^/L]	32.2 (2.4-171.4)	38.0 (0.9-274.6)	0.35
Hemoglobin [median (range); g/dL]	12.9 (8.2-16.8)	12.8 (8.9-16.8)	0.97
Platelet count [median (range); x10^9^/L]	136.0 (49.0-412.0)	149.5 (52.0-292.0)	0.64
Clinical Stage (Rai) 0 I - II III-IV	0 (0%) 21 (63.6%) 12 (36.4%)	3 (9.4%) 17 (53.1%) 12 (37.5%)	0.24
Bulky nodes (largest node >5 cm)^1^	11 (33.3%)	10 (31.3%)	1.00
Beta-2-microglobulin [median (range); g/L] ≥4 g/L	3.6 (0.3-9.0) 14 (42.4%)	3.8 (0.3-10.0) 13 (40.6%)	0.74 1.00
ZAP-70 >20%	17 (51.5%)	17 (53.1%)	1.00
CD38 >30%	12 (36.4%)	18 (56.3%)	0.14
IGHV^2^ unmutated (<2%)	11 (40.7%)	17 (63.0%)	0.17
CD49 >45%	13 (39.4%)	18 (56.3%)	0.45
FISH hierarchy del (13q14.2) Trisomy 12 Normal del11q23 del17p13 6q- Other^3^	8 (25.8%) 6 (19.4%) 10 (32.3%) 4 (12.9%) 1 (3.2%) 2 (6.5%) 2 (6.5%)	2 (6.3%) 7 (21.9%) 9 (28.1%) 4 (12.5%) 2 (6.3%) 0 (0.0%) 1 (3.1%)	1.00

PCR, pentostatin, cyclophosphamide, and rituximab; B, bevacizumab; ALC, absolute lymphocyte count; *IGHV*, immunoglobulin heavy chain variable region gene; FISH, fluorescence in situ hybridization

1 By CT **scan**

2 *IGHV* could not be determined in 6 patients from PCR-B and 5 patients from PCR. These patients were excluded in the calculation of the percentage for *IGHV* mutational status.

3 Two patients on Arm A are reported under ‘other’ FISH category. One patient reported a 13q14 = 89%, Ig 1 = 40%, and one patient reported a partial deletion of the 51 lgH region. One patient from Arm B had 8q24.1 (MYCx3).

### Toxicity

Patients received a median of 6 cycles (range, 1-6 cycles) in each arm. Twenty-seven (81.8%) patients completed the intended 6 cycles of PCR-B on Arm A, and 27 (84.4%) 6 cycles of PCR on Arm B. Reasons for treatment discontinuation before completion of 6 cycles of PCR-B were patient choice, infectious complication (pneumonia) and cardiovascular complications (hypertension, myocardial ischemia, congestive heart failure, and aortic dissection); reasons for treatment discontinuation of PCR were pneumonia, nausea, neurological symptoms, and treating physician choice. During treatment, 7 patients experienced a dose delay (1 on PCR-B [Arm A] and 6 on PCR [Arm B]), and 1 patient (Arm B) required a dose reduction in pentostatin and cyclophosphamide. Three patients on PCR-B required temporary omission of Bevacizumab due to hypertension, need for polypectomy, and proteinuria (in the latter case, it was discontinued at cycle 5, and not resumed later). Five patients did not receive Bevacizumab on day 43 of cycle 6: 2 due to patient decision, one missed in error, one febrile neutropenia, and one central nervous system hemorrhage. One patient in Arm B required temporary omission of rituximab due to cytokine release syndrome.

Grade 3+ adverse events observed during treatment in both arms are reported in Table 2. Twenty-three of 33 patients (69.7%) on PCR-B and 14 of 32 patients (44%) on PCR experienced at least one grade 3+ event at least possibly related to treatment (p=0.05). Nine of 33 patients (27.3%) on PCR-B and 5 of 32 patients (15.6%) on PCR experienced a grade 4+ event at least possibly related to treatment (p=0.37). Grade 3-4 cardiovascular toxicity was present in 11 patients from PCR-B (7 cases of hypertension, one myocarditis, one left ventricular dysfunction, one left ventricular failure, and one with Torsade de Pointes with left ventricular failure) and one patient from PCR (hypertension) (33% vs. 3%, p<0.003).

**Table 2:** Grade 3+ toxicity at least possibly related to treatment

CTCAE Classification	PCR Arm B (*N*= 32)	PCR-B Arm A (*N*= 33)
	Grade 3+	Grade 3+
Heme toxicity (grade 3+)	10 (31.3%)	12 (36.4%)
Non-heme toxicity (grade 3+)	9 (28.1%)	18 (54.5%)
Neutropenia	7 (21.9%)	9 (27.3%)
Thrombocytopenia^1^	1 (3.1%)	5 (15.1%)
Hemoglobin decrease^1^	2 (6.3%)	1 (3.0%)
Dyspnea	0	3 (9.1%)
Left ventricular failure	0	2 (6.1%)
Left ventricular dysfunction	0	1 (3.0%)
Sepsis	1 (3.1%)	1 (3.0%)
Fatigue	0	1 (3.0%)
Hypertension	1 (3.1%)	7 (21.2%)
Headache	0	1 (3.0%)
Confusion	1 (3.1%)	0
Depressed consciousness	1 (3.1%)	0
Neurological decline^4^	1 (3.1%)	0
Protein Urine Positive	0	2 (6.1%)
Creatinine increase	1 (3.1%)	2 (6.1%)
Nausea	1 (3.1%)	1 (3.0%)
Cystitis	0	1 (3.0%)
Ascites	0	1 (3.0%)
Bladder hemorrhage	0	1 (3.0%)
Bladder pain	0	1 (3.0%)
Blood disorder	0	1 (3.0%)
Cough	0	1 (3.0%)
Myocarditis	0	1 (3.0%)
Dehydration	0	1 (3.0%)
Renal Failure	1 (3.1%)	1 (3.0%)
Serum sodium increase	0	1 (3.0%)
Serum sodium decrease	1 (3.1%)	0
Vomiting	1 (3.1%)	2 (6.1%)
Torsade de pointes	0	1 (3.0%)
Vascular disorder	0	1 (3.0%)
Fever	1 (3.1%)	0
Blood glucose increase	1 (3.1%)	0
Diarrhea	1 (3.1%)	0
Hypersensitivity	1 (3.1%)	0
Intracranial Hemorrhage	0	1 (3.0%)
Febrile Neutropenia	0	1 (3.0%)

### Treatment response

All 65 eligible patients were evaluable for response after therapy. On the PCR-B arm, all 33 patients had a response (100% overall response rate), with 18 patients (54.5%) having a CR or CR with incomplete marrow recovery (CR-i). On the PCR arm, 31 patients had a response (96.9% overall response rate), with 10 patients (31.3%) evaluated as a CR or CR-i (p=0.08). The > 4 patient difference in CR/CR-i between arms met the protocol specified criteria to select PCR-B as the recommended arm for further study.

A MRD-negative remission was achieved at the end of treatment in 7/21 (33.3%) patients on PCR-B, and 5/22 (22.7%) on PCR (Table 3). There was no statistically significant relationship between any baseline prognostic characteristic and achievement of CR/CR-i or MRD eradication in the PCR-B arm.

**Table 3:** Response to therapy

	PCR-B (Arm A) (*N*= 33)	PCR (Arm B) (*N*= 32)	*p*-value
ORR	33 (100%)	31 (96.9%)	0.49
CR/CR-i	18 (54.5%)	10 (31.3%)	0.08
CCR	3 (9.1%)	3 (9.4%)	1.00
n-PR	5 (15.2%)	13 (40.6%)	0.028
PR	7 (21.2%)	5 (15.6%)	0.75
SD	0 (0.0%)	0 (0.0%)	NA
NE	0 (0.0%)	1(3.0%)	0.49
CR/n-PR with MRD negative	7/21 (33.3%)	5/22 (22.7%)	0.51

ORR, overall response rate; CR, complete remission; CR-i, CR with incomplete bone marrow recovery; CCR, clinical CR; PR, partial remission; n-PR, nodular PR; SD, stable disease; MRD, minimal residual disease; NE, not evaluated

^1^MRD assays were preferentially performed on bone marrow aspirate with use of peripheral blood if bone marrow aspirate was not available.

### Survival

**Figure 1:**
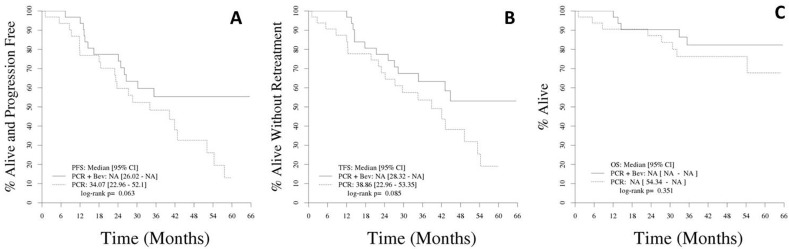
**A.** Progression-free survival. **B.** Treatment-free survival. **C.** Overall survival

After a median follow-up among living patients of 46 months (range, 2-66), 12 patients (36.4%) on PCR-B and 17 (53.1%) on PCR have progressed. The median PFS has not yet been reached for PCR-B (Arm A) and was 34 months (95% confidence interval [CI], 23-52) for PCR alone (Arm B, p=0.06; Figure 1A). Twenty-four patients have required salvage therapy, 10 (30%) on PCR-B and 14 (44%) on PCR. Median TFS has not yet been reached for PCR-B (Arm A, 95% CI, 28 - not reached) and was 39 months (95% CI, 23 - 53) for PCR alone (Arm B, p=0.09; Figure 1B). As of last follow-up, 13 patients have died; 5 on PCR-B (2 from progressive disease, 1 from hemolytic anemia, 1 from sepsis, and one from unknown causes) and 8 on PCR (2 from disease progression, 2 from second cancers, 1 from congestive heart failure, 1 from sepsis, 1 in a car accident, and one from unknown cause). Median OS has not been reached in either arm (p=0.35; Figure 1C).

**Figure 2:**
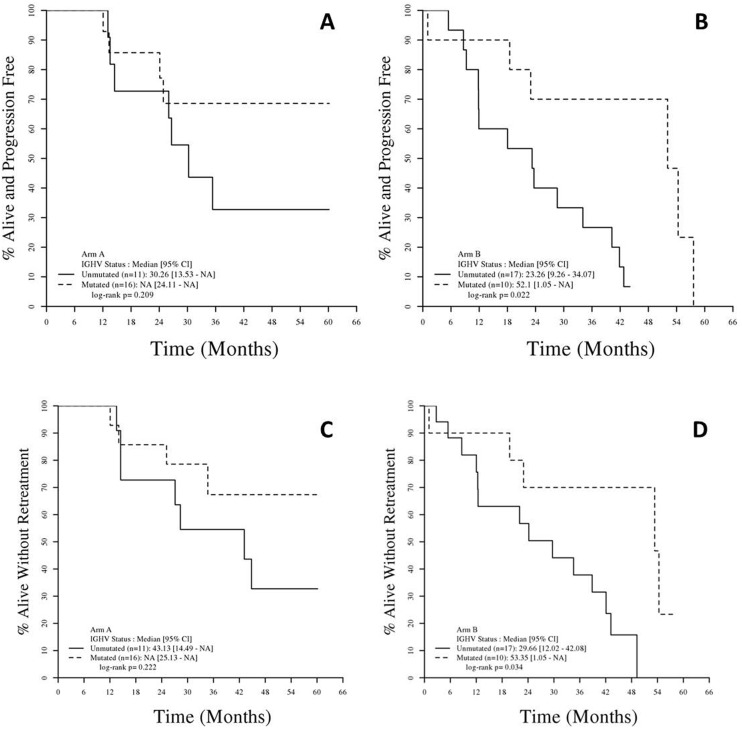
Progression Free Survival (PFS) and Treatment-free survival (TFS) and by *IGHV* mutational status

While unmutated *IGHV* status determined a shorter PFS (p=0.02) and TFS (p=0.03) in the arm B, it did not affect survival when bevacizumab was added to PCR (Figure 2).

### Kinetics of plasma angiogenic and chemokine cytokine levels

Plasma sample for evaluation of angiogenic cytokines were available for 50 patients (25 on Arm A and 25 on Arm B). Median levels of VEGF, b-FGF, TSP-1, CCL-3 and CCL-4 at baseline and at time of response assessment for each arm are shown in Table 4.

**Table 4:** Plasma cytokine kinetics in the 2 arms

Arm	Cytokine	Number of Samples	Baseline Median (Min, Max)	Response Median (Min, Max)	Change from Baseline Median (Min, Max)	*p*-value Baseline*vs*. Response1	*p*-value Change from Baseline Arm A*vs*. Arm B2
A	VEGF	25	29.77	57.05	18.24	0.00002	0.00001
			(9.58, 83.92)	(12.60, 99.85)	(−56.31, 66.56)		
B	VEGF	25	28.82	27.72	−2.65	0.23795	
			(12.35, 15.08)	(7.21, 57.96)	(−84.61, 33.89)		
A	TSP1	25	9272	8296	−112	0.804	0.12061
			(2928, 23552)	(3783, 22478)	(−7023, 7261)		
B	TSP1	25	7679	8841	1018	0.01163	
			(449, 1211)	(5375, 21159)	(−3441, 15185)		
A	CCL3	25	85.55	79.14	−8.89	0.00002	0.85368
			(21.65,561.7)	(21.65, 162.66)	(−399.08, 12.57)		
B	CCL3	25	93.21	76.17	−10.68	0.00006	
			(21.65,7265.4)	(21.65, 344.54)	(−7185.11, 96.93)		
A	CCL4	25	151.3	81.21	−39.66	0.00009	0.37211
			(50.2,3975.8)	(41.45, 230.00)	(−3811.16, 52.05)		
B	CCL4	25	121.4	65.77	−51.93	<0.00001	
			(39.7, 5620.7)	(28.45, 657.97)	(−5486.09, 19.00)		
A	FGFb	25	27.18	27.02	3.59	0.65835	0.27677
			(2.74, 464.28)	(2.74 66.38)	(−397.90, 37.50)		
B	FGFb	25	28.11	27.18	−4.01	0.0333	
			(2.74, 384.40)	(2.74, 184.41)	(−349.65, 138.84)		

^1^Signed Rank *p*-value; ^2^Rank-sum *p*-value

**Figure 3:**
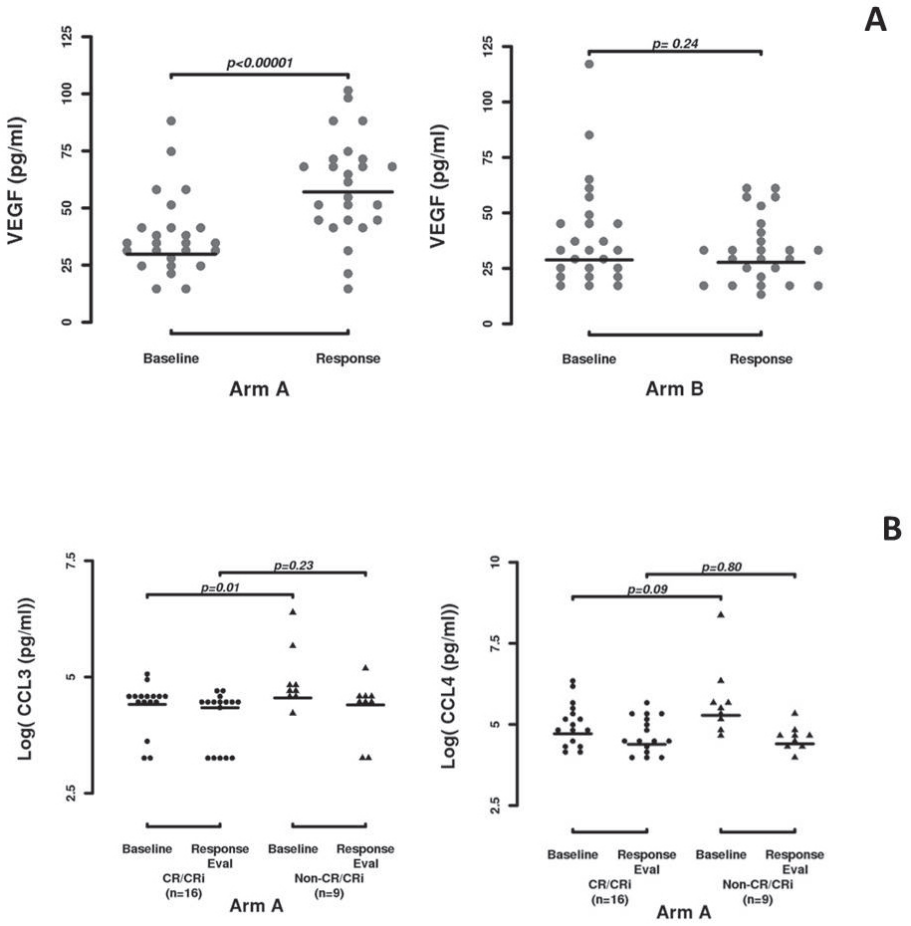
Plasma cytokine kinetics **A.** VEGF kinetics in arm A and arm B. **B.** CCL-3 and CCL-4 kinetics in the PCR-B arm, comparing responders to non-responders.

Compared to baseline, a significant increase in VEGF levels was observed at the time of response assessment for patients treated with PCR-B (Arm A, p<0.001). No such change was observed for patients treated with PCR only (comparison arm A to arm B p<0.001)(Figure 3A). Although statistically significant changes in CCL-3, CCL-4, TSP-1 and/or b-FGF were observed in one arm or the other, no statistically significant difference was observed between arms (Table 4).

In the PCR-B arm, there was a significant difference in baseline values of CCL-3 between patients that achieved a CR/CR-i vs. those that did not (median 82.3 vs. median 94.6, respectively; p=0.01) and a trend for association was observed for baseline CCL-4 levels (p=0.09). However, no differences in post-treatment values were observed (Figure 3B). No significant correlations were found between cytokine plasma levels and responses for patients treated in the PCR only arm.

## DISCUSSION

We report here the first randomized trial combining anti-VEGF therapy with CIT for patients with previously untreated CLL.

The addition of bevacizumab to chemoimmunotherapy was found to be safe, with no significant differences in severe adverse events between the 2 arms, other than an increase in non-fatal cardiovascular complications (33% vs 3%, p<0.003); none of the 7 cases of hypertension presented as an hypertensive emergency, and they all resolved with non-urgent medical management; the 2 cases of CHF and the 1 case of myocarditis all occurred in patients with pre-existing heart dysfunction who had discontinued their home medications, and resolved with non-urgent medical management; the only grade 4 cardiovascular complication was a torsade de pointe, which occurred as a peri-surgical complication in a patient with pre-existing aortic aneurysm, developing an aortic dissection requiring surgical intervention.

Patients treated with PCR in combination with anti-VEGF therapy had a trend toward higher complete remission rate (54.5%) than patients receiving PCR alone (31.3%). This difference met the protocol specified criteria to select PCR-B as the arm recommended for further evaluation. A higher (though not significant) MRD eradication rate was observed with the addition of bevacizumab, translating into longer PFS and TFS. In addition, when bevacizumab was added to CIT, the difference in median PFS and TFS by *IGHV* mutational status observed with CIT only was not observed; CIT can achieve long-term disease-free survival in patients with mutated *IGHV*, while only newer biological agents have so far achieved similar results in patient with unmutated *IGHV*.[27–30]

How do our findings relate to previous trials of anti-VEGF therapy in CLL? We have conducted separate phase II trials for three separate anti-VEGF therapies for patients with relapsed/refractory CLL.[14] In total 46 patients were accrued to trials of single-agent anti-VEGF antibody and there was no sign of efficacy and no CRs or PRs were noted. Wierda et al reported a phase 2 study of bevacizumab in combination with FCR in patients with relapsed refractory CLL.[31] This approach did not clearly show an advantage relative to historical trials of FCR alone. Patients with relapsed, refractory disease are a distinct clinical population and recent genetic studies have pointed out that leukemic cells that have survived or expanded after the selective pressure of prior therapy are likely more fit to survive in the host microenvironment based on clonal evolution and changes in clonal architecture when compared to treatment naïve patients.[32–34]

The most prominent toxicity noted with PCR-B was cardiovascular toxicity. Cardiovascular toxicity is a well-recognized complication of bevacizumab due to its endothelial effects. Indeed, the most common complications described with its use, both as single agent or in combination with chemotherapy, are hypertension, congestive heart failure, and arterial and venous thromboembolic disease. The incidence of such complications in our study was not increased, when compared to other published experiences employing this agent.[35] In addition despite the occurrence of cardiovascular toxicities on the PCR-B arm an equivalent number of CLL patients (~80%) were able to complete the prescribed 6 cycles for both arms.

Although we found no association between baseline traditional prognostic factors and achievement of CR among patients treated with PCR-B, there were both expected and unexpected findings derived from the analysis of the microenvironment cytokine kinetics. As anticipated we found that lower baseline CCL-3 plasma levels were significantly associated with higher response rate when bevacizumab was added to chemoimmunotherapy. CCL-3 and CCL-4, previously called macrophage inflammatory protein-1a (MIP-1a) and MIP-1b, are chemokines of the CC sub-family, inducible in many hematopoietic cells, such as macrophages and dendritic cells, but also B and T lymphocytes. [36] Indeed CLL cells secrete these 2 chemokines in response to BCR stimulation, to attract accessory cells and enhance their microenvironment and this activity can be a candidate for a distinct disease progression event.[37, 38] Other studies have convincingly demonstrated that baseline CCL-3 levels associate with survival in CLL and other B-cell malignancy, in concordance with our results.[39, 40]

CCL-3 and CCL-4 reduction in plasma levels is typically observed in response to BCR inhibitors, such as Ibrutinib or Idelalisib,[41, 42] but there are no *in-vitro* or *in-vivo* data to suggest a direct interaction between bevacizumab and the BCR. It is important to notice that in our study, despite the specific prognostic role played by CCL-3 and CCL-4 levels in the PCR-B arm, there was no significant difference in their post-treatment decrease between the 2 arms. This may suggest that the reduction of CCL-3 and CCL-4, mediated by chemoimmunotherapy, is not directly responsible for the observed positive clinical results observed with the PCR-B arm, but rather suggest the influence of bevacizumab is an important regulator of the CLL disease process.

The major clinical question is does any anti-VEGF approach in CLL also require the use of an additional powerful component like CIT? The paradoxical post-treatment increase in VEGF levels observed in our study may have been temporary and preceding a following steady decrease, as already observed in other studies where therapeutic agents modulating the microenvironment are employed.[43–45] Unfortunately, we were not able to discern if this occurred as in this study VEGF levels were only done at baseline and at the time of response and not re-measured beyond the end-of-treatment assessment.

In conclusion, the addition of bevacizumab to chemoimmunotherapy is safe and effective. Although cardiovascular toxicity affected approximately 30% of PCR-B patients, it was manageable and did not impact patients' ability to complete therapy. Even with the advent of very effective novel single agent signal inhibitor therapy, CIT remains the standard of care for patients with previously untreated CLL particularly those with mutated IGVH status.[1, 46] The addition of anti-VEGF therapy to CIT did produce higher complete response rates and resulted in longer progression-free and treatment-free survival, when compared to chemoimmunotherapy alone, independently from *IGHV* mutational status. Given the recent dramatic clinical impact of signal inhibitors such as ibrutinib or Idelalisib to induce high ORRs of very long duration in relapsed/refractory CLL[47] and our findings that anti-VEGF addition to CIT look promising in terms of clinical outcome for upfront CLL, it is tempting to speculate that combinations of signal inhibitors and anti-VEGF agents should be tested.

## MATERIALS AND METHODS

### Patient eligibility

Eligible patients were previously untreated and had CLL in need of treatment according to the NCI-WG criteria.[19] Patients were required to have an ECOG performance status of 0 to 2, and have adequate renal and hepatic function. Individuals with recent (<1 month) myocardial infarction, class III or IV heart failure, uncontrolled infection, infection with human immunodeficiency virus (HIV), active hepatitis B or C infection, or active hemolytic anemia were excluded. Patients with other malignancies were allowed to participate, provided they were not receiving treatment and had a life expectancy >2 years. There was no upper age limit on eligibility. The protocol was reviewed and approved by the Mayo Clinic institutional review boards and conducted in accordance to the Declaration of Helsinki. It was registered with clinicaltrials.gov (identifier NCT00816595).

### Treatment plan and toxicity evaluation

After providing written informed consent, all patients were offered 6 cycles of pentostatin (2 mg/m^2^ on day 1), cyclophosphamide (600 mg/m^2^ on day 1), and rituximab (cycle 1: 100 mg on day 1, 375 mg/m^2^ on day 2; cycles 2-6: 375 mg/m^2^ on day 1) given intravenously every 21 days. This combination has been shown to be an effective regimen for both previously treated [20, 21] and therapy-naïve [22–25] CLL patients.[26]

Patients were randomized to receive either PCR in combination with bevacizumab (PCR-B; Arm A) or PCR alone (Arm B) using a dynamic allocation procedure incorporating stratification based on Rai stage (0-II vs III-IV) and their FISH prognosis group [favorable (normal, +12, 13q-, other) vs. unfavorable (17p- or 11q-)]. Patients randomized to arm A received PCR in combination with bevacizumab (15 mg/Kg on day 1 of each cycle, and then on day 22 and 43 of cycle 6) administered intravenously every 21 days. Patients on arm B received PCR alone. Prophylaxis against Pneumocystis jiroveci (sulfamethoxazole-trimethoprim or alternative) and herpes zoster (valacyclovir or alternative) were given to all patients for 1 year from the start of cycle 1. All patients were given allopurinol (300 mg orally once daily) on days 1 through 14 of cycle 1. Pegfilgrastim was administered on day 2 of each cycle.

Platelet and hemoglobin adverse events were graded according to the IWCLL CLL Working Group grading scale for hematologic toxicity.[19] All other adverse events were graded according to the NCI Common Toxicity Criteria (version 4). Toxicity was defined as an adverse event that is possibly, probably, or definitely related to treatment.

### Response assessment

Patients completing 6 cycles of therapy underwent complete restaging including evaluation for minimal residual disease (MRD) using flow cytometry (assay sensitivity <0.01%; 500,000 events collected). MRD assessment was limited to patients with a complete response (CR)(34) or a nodular partial remission (NPR)(18). Restaging occurred 12 weeks after day 1 of cycle 6. MRD assays were preferentially performed on bone marrow aspirate with use of peripheral blood if bone marrow aspirate was not available (n=2). Responses were graded according to the NCI/IWCLL Working Group criteria.[19] Bone marrow biopsies were performed at registration and at response evaluation to document complete response. Although primary response categorization was performed by physical exam in accord with the iwCLL criteria,[19] computed tomography (CT) scans of the chest, abdomen, and pelvis were also performed in all patients at registration and at the response evaluation. A patient was considered evaluable for response if they were eligible and initiated treatment.

### Correlative studies

To evaluate the association between angiogenic and chemokine factors and depth of response, we examined serum levels of VEGF, b-FGF, anti-[thrombospondin (TSP)-1], chemokine ligand (CCL)-3 and CCL-4 for each patient immediately prior to treatment and at time of response assessment. VEGF (isoform 165), b-FGF, CCL-3 and CCL-4 were measured using Quantikine kits (R&D Systems, Minneapolis, MN, USA) and TSP using the Accucyte assay (CytImmuneSciences Inc, Rockville, MD, USA) according to the manufacturer's instructions. Other molecular and biological prognostic parameters, including CD38, ZAP-70, *IGHV* mutation status, and recurrent cytogenetic abnormalities as assessed by FISH, were performed on baseline study samples from the patient cohort as previously reported.[25]

### Statistical methods

This study utilized a randomized phase II flexible screening design which required a total of 62 evaluable patients (31 patients per arm). The primary endpoint of the trial was the rate of complete response. One regimen would have been identified as the more promising of the two if the difference in the number of patients with a CR was at least 4 of 31 patients. This study design has at least 80% power to select the correct regimen to bring forward into larger and confirmatory studies.

Protocol specified secondary endpoints included the rate of MRD negative remissions for each arm and assessment of whether molecular prognostic parameters (e.g. *IGHV*, FISH, ZAP-70, and CD38) predicted response. Progression-free survival (PFS) was defined as the time from registration to disease progression or death due to any cause. Treatment-free survival (TFS) was defined as time from registration to initiation of subsequent treatment for CLL or death due to any cause. Overall survival (OS) was defined as the time from registration to death due to any cause. The distributions of time to event measures were estimated using the Kaplan-Meier method, and differences between groups were evaluated by log-rank statistics. Clinical characteristics and prognostic factors were compared between groups using Fisher's exact test and Wilcoxon's rank-sum test for categorical and continuous factors, respectively. Wilcoxon's signed rank test was used to compare cytokine values across time.

## References

[1] Hallek M, Fischer K, Fingerle-Rowson G, Fink AM, Busch R, Mayer J, Hensel M, Hopfinger G, Hess G, von Grunhagen U, Bergmann M, Catalano J, Zinzani PL (2010). Addition of rituximab to fludarabine and cyclophosphamide in patients with chronic lymphocytic leukaemia: a randomised, open-label, phase 3 trial. Lancet.

[2] Goede V, Fischer K, Busch R, Jaeger U, Dilhuydy MS, Wickham N, De Guibert S, Ritgen M, Langerak AW, Bieska G, Engelke A, Humphrey K, Wenger M (2013). Chemoimmunotherapy with GA101 plus chlorambucil in patients with chronic lymphocytic leukemia and comorbidity: results of the CLL11 (BO21004) safety run-in. Leukemia.

[3] Bottcher S, Ritgen M, Fischer K, Stilgenbauer S, Busch RM, Fingerle-Rowson G, Fink AM, Buhler A, Zenz T, Wenger MK, Mendila M, Wendtner CM, Eichhorst BF (2012). Minimal residual disease quantification is an independent predictor of progression-free and overall survival in chronic lymphocytic leukemia: a multivariate analysis from the randomized GCLLSG CLL8 trial. Journal of clinical oncology.

[4] Ten Hacken E, Burger JA (2015). Microenvironment interactions and B-cell receptor signaling in Chronic Lymphocytic Leukemia: Implications for disease pathogenesis and treatment. Biochimica et biophysica acta.

[5] Lee YK, Bone ND, Strege AK, Shanafelt TD, Jelinek DF, Kay NE (2004). VEGF receptor phosphorylation status and apoptosis is modulated by a green tea component, epigallocatechin-3-gallate (EGCG), in B-cell chronic lymphocytic leukemia. Blood.

[6] Lee YK, Shanafelt TD, Bone ND, Strege AK, Jelinek DF, Kay NE (2005). VEGF receptors on chronic lymphocytic leukemia (CLL) B cells interact with STAT 1 and 3: implication for apoptosis resistance. Leukemia.

[7] Shanafelt TD, Kay NE (2006). The clinical and biologic importance of neovascularization and angiogenic signaling pathways in chronic lymphocytic leukemia. Seminars in oncology.

[8] Kay NE (2004). The angiogenic status of B-CLL B cells: role of the VEGF receptors. Leukemia Research.

[9] Long BW, Witte PL, Abraham GN, Gregory SA, Plate JM (1995). Apoptosis and interleukin 7 gene expression in chronic B-lymphocytic leukemia cells. Proceedings of the National Academy of Sciences of the United States of America.

[10] Farahani M, Treweeke AT, Toh CH, Till KJ, Harris RJ, Cawley JC, Zuzel M, Chen H (2005). Autocrine VEGF mediates the antiapoptotic effect of CD154 on CLL cells. Leukemia.

[11] Ghosh AK, Shanafelt TD, Cimmino A, Taccioli C, Volinia S, Liu CG, Calin GA, Croce CM, Chan DA, Giaccia AJ, Secreto C, Wellik LE, Lee YK (2009). Aberrant regulation of pVHL levels by microRNA promotes the HIF/VEGF axis in CLL B cells. Blood.

[12] Bogusz J, Majchrzak A, Medra A, Cebula-Obrzut B, Robak T, Smolewski P (2013). Mechanisms of action of the anti-VEGF monoclonal antibody bevacizumab on chronic lymphocytic leukemia cells. Postepy higieny i medycyny doswiadczalnej.

[13] Kay NE, Bone ND, Tschumper RC, Howell KH, Geyer SM, Dewald GW, Hanson CA, Jelinek DF (2002). B-CLL cells are capable of synthesis and secretion of both pro- and anti-angiogenic molecules. Leukemia.

[14] Shanafelt T, Zent C, Byrd J, Erlichman C, Laplant B, Ghosh A, Call T, Villalona-Calero M, Jelinek D, Bowen D, Laumann K, Wu W, Hanson C (2010). Phase II trials of single-agent anti-VEGF therapy for patients with chronic lymphocytic leukemia. Leukemia & Lymphoma.

[15] Hurwitz H, Fehrenbacher L, Novotny W, Cartwright T, Hainsworth J, Heim W, Berlin J, Baron A, Griffing S, Holmgren E, Ferrara N, Fyfe G, Rogers B (2004). Bevacizumab plus irinotecan, fluorouracil, and leucovorin for metastatic colorectal cancer. The New England journal of medicine.

[16] Miller K, Wang M, Gralow J, Dickler M, Cobleigh M, Perez EA, Shenkier T, Cella D, Davidson NE (2007). Paclitaxel plus bevacizumab versus paclitaxel alone for metastatic breast cancer. The New England journal of medicine.

[17] Sandler A, Gray R, Perry MC, Brahmer J, Schiller JH, Dowlati A, Lilenbaum R, Johnson DH (2006). Paclitaxel-carboplatin alone or with bevacizumab for non-small-cell lung cancer. The New England journal of medicine.

[18] Shanafelt TD, Byrd JC, La PB, Zent CS, Call T, Secreto C, Grever MR, Lin TS, Kay NE (2009). Pretreatment angiogenic cytokines predict response to chemoimmunotherapy in patients with chronic lymphocytic leukaemia. British journal of haematology.

[19] Hallek M, Cheson BD, Catovsky D, Caligaris-Cappio F, Dighiero G, Dohner H, Hillmen P, Keating MJ, Montserrat E, Rai KR, Kipps TJ (2008). Guidelines for the diagnosis and treatment of chronic lymphocytic leukemia: a report from the International Workshop on Chronic Lymphocytic Leukemia updating the National Cancer Institute-Working Group 1996 guidelines. Blood.

[20] Weiss MA, Maslak PG, Jurcic JG, Scheinberg DA, Aliff TB, Lamanna N, Frankel SR, Kossman SE, Horgan D (2003). Pentostatin and cyclophosphamide: an effective new regimen in previously treated patients with chronic lymphocytic leukemia. Journal of clinical oncology.

[21] Lamanna N, Kalaycio M, Maslak P, Jurcic JG, Heaney M, Brentjens R, Zelenetz AD, Horgan D, Gencarelli A, Panageas KS, Scheinberg DA, Weiss MA (2006). Pentostatin, cyclophosphamide, and rituximab is an active, well-tolerated regimen for patients with previously treated chronic lymphocytic leukemia. Journal of clinical oncology.

[22] Shanafelt TD, Lin T, Geyer SM, Zent CS, Leung N, Kabat B, Bowen D, Grever MR, Byrd JC, Kay NE (2007). Pentostatin, cyclophosphamide, and rituximab regimen in older patients with chronic lymphocytic leukemia. Cancer.

[23] Dillman RO, Schreeder MT, Hon JK, Connelly EF, DePriest C, Cutter K (2007). Community-based phase II trial of pentostatin, cyclophosphamide, and rituximab (PCR) biochemotherapy in chronic lymphocytic leukemia and small lymphocytic lymphoma. Cancer biotherapy & radiopharmaceuticals.

[24] Kay NE, Shanafelt TD, Byrd JC, Grever MR (2007). Community-based phase II trial of PCR for CLL/SLL patients. Cancer biotherapy & radiopharmaceuticals.

[25] Kay NE, Geyer SM, Call TG, Shanafelt TD, Zent CS, Jelinek DF, Tschumper R, Bone ND, Dewald GW, Lin TS, Heerema NA, Smith L, Grever MR (2007). Combination chemoimmunotherapy with pentostatin, cyclophosphamide, and rituximab shows significant clinical activity with low accompanying toxicity in previously untreated B chronic lymphocytic leukemia. Blood.

[26] Reynolds C, Di Bella N, Lyons RM, Hyman W, Richards DA, Robbins GJ, Vellek M, Boehm KA, Zhan F, Asmar L (2012). A Phase III trial of fludarabine, cyclophosphamide, and rituximab vs. pentostatin, cyclophosphamide, and rituximab in B-cell chronic lymphocytic leukemia. Investigational new drugs.

[27] Thompson PA, Tam CS, O'Brien SM, Wierda WG, Stingo F, Plunkett W, Smith SC, Kantarjian HM, Freireich EJ, Keating MJ (2015). Fludarabine, cyclophosphamide and rituximab achieves long-term disease-free survival in IGHV-mutated chronic lymphocytic leukemia. Blood.

[28] Rossi D, Terzi-di-Bergamo L, De Paoli L, Cerri M, Ghilardi G, Chiarenza A, Bulian P, Visco C, Mauro FR, Morabito F, Cortelezzi A, Zaja F, Forconi F (2015). Molecular prediction of durable remission after first-line fludarabine-cyclophosphamide-rituximab in chronic lymphocytic leukemia. Blood.

[29] Fischer K, Bahlo J, Fink AM, Goede V, Herling CD, Cramer P, Langerbeins P, von Tresckow J, Engelke A, Maurer C, Kovacs G, Herling M, Tausch E (2015). Long term remissions after FCR chemoimmunotherapy in previously untreated patients with CLL: updated results of the CLL8 trial. Blood.

[30] Guo A, Lu P, Galanina N, Nabhan C, Smith SM, Coleman M, Wang YL (2015). Heightened BTK-dependent cell proliferation in unmutated chronic lymphocytic leukemia confers increased sensitivity to ibrutinib. Oncotarget.

[31] Jain P, Lee HJ, Qiao W, Wierda W, Benjamini O, Burger J, Ferrajoli A, Estrov Z, Kantarjian H, Keating M, O'Brien S (2014). FCR and bevacizumab treatment in patients with relapsed chronic lymphocytic leukemia. Cancer.

[32] Ojha J, Ayres J, Secreto C, Tschumper R, Rabe K, Van Dyke D, Slager S, Shanafelt T, Fonseca R, Kay NE, Braggio E (2015). Deep sequencing identifies genetic heterogeneity and recurrent convergent evolution in chronic lymphocytic leukemia. Blood.

[33] Landau DA, Tausch E, Taylor-Weiner AN, Stewart C, Reiter JG, Bahlo J, Kluth S, Bozic I, Lawrence M, Bottcher S, Carter SL, Cibulskis K, Mertens D (2015). Mutations driving CLL and their evolution in progression and relapse. Nature.

[34] Landau DA, Carter SL, Stojanov P, McKenna A, Stevenson K, Lawrence MS, Sougnez C, Stewart C, Sivachenko A, Wang L, Wan Y, Zhang W, Shukla SA (2013). Evolution and impact of subclonal mutations in chronic lymphocytic leukemia. Cell.

[35] Economopoulou P, Kotsakis A, Kapiris I, Kentepozidis N (2015). Cancer therapy and cardiovascular risk: focus on bevacizumab. Cancer management and research.

[36] Eberlein J, Nguyen TT, Victorino F, Golden-Mason L, Rosen HR, Homann D (2010). Comprehensive assessment of chemokine expression profiles by flow cytometry. The Journal of clinical investigation.

[37] Burger JA, Quiroga MP, Hartmann E, Burkle A, Wierda WG, Keating MJ, Rosenwald A (2009). High-level expression of the T-cell chemokines CCL3 and CCL4 by chronic lymphocytic leukemia B cells in nurselike cell cocultures and after BCR stimulation. Blood.

[38] Castellino F, Huang AY, Altan-Bonnet G, Stoll S, Scheinecker C, Germain RN (2006). Chemokines enhance immunity by guiding naive CD8+ T cells to sites of CD4+ T cell-dendritic cell interaction. Nature.

[39] Sivina M, Hartmann E, Kipps TJ, Rassenti L, Krupnik D, Lerner S, LaPushin R, Xiao L, Huang X, Werner L, Neuberg D, Kantarjian H, O'Brien S (2011). CCL3 (MIP-1alpha) plasma levels and the risk for disease progression in chronic lymphocytic leukemia. Blood.

[40] Takahashi K, Sivina M, Hoellenriegel J, Oki Y, Hagemeister FB, Fayad L, Romaguera JE, Fowler N, Fanale MA, Kwak LW, Samaniego F, Neelapu S, Xiao L (2015). CCL3 and CCL4 are biomarkers for B cell receptor pathway activation and prognostic serum markers in diffuse large B cell lymphoma. British journal of haematology.

[41] Ponader S, Chen SS, Buggy JJ, Balakrishnan K, Gandhi V, Wierda WG, Keating MJ, O'Brien S, Chiorazzi N, Burger JA (2012). The Bruton tyrosine kinase inhibitor PCI-32765 thwarts chronic lymphocytic leukemia cell survival and tissue homing in vitro and in vivo. Blood.

[42] Hoellenriegel J, Meadows SA, Sivina M, Wierda WG, Kantarjian H, Keating MJ, Giese N, O'Brien S, Yu A, Miller LL, Lannutti BJ, Burger JA (2011). The phosphoinositide 3′-kinase delta inhibitor, CAL-101, inhibits B-cell receptor signaling and chemokine networks in chronic lymphocytic leukemia. Blood.

[43] Strati P, Keating MJ, Wierda WG, Badoux XC, Calin S, Reuben JM, O'Brien S, Kornblau SM, Kantarjian HM, Gao H, Ferrajoli A (2013). Lenalidomide induces long-lasting responses in elderly patients with chronic lymphocytic leukemia. Blood.

[44] Ebos JM, Lee CR, Christensen JG, Mutsaers AJ, Kerbel RS (2007). Multiple circulating proangiogenic factors induced by sunitinib malate are tumor-independent and correlate with antitumor efficacy. Proceedings of the National Academy of Sciences of the United States of America.

[45] Bilen MA, Zurita AJ, Ilias-Khan NA, Chen HC, Wang X, Kearney AY, Hodges S, Jonasch E, Huang S, Khakoo AY, Tannir NM (2015). Hypertension and Circulating Cytokines and Angiogenic Factors in Patients With Advanced Non-Clear Cell Renal Cell Carcinoma Treated With Sunitinib: Results From a Phase II Trial. The oncologist.

[46] Keating MJ, O'Brien S, Albitar M, Lerner S, Plunkett W, Giles F, Andreeff M, Cortes J, Faderl S, Thomas D, Koller C, Wierda W, Detry MA (2005). Early results of a chemoimmunotherapy regimen of fludarabine, cyclophosphamide, and rituximab as initial therapy for chronic lymphocytic leukemia. Journal of clinical oncology.

[47] Brower V (2015). Targeted Therapies Improve Outlook for Chronic Lymphocytic Leukemia. Journal of the National Cancer Institute.

